# Rolling vs. Swing: A Strategy for Enhancing Locomotion Speed and Stability in Legged Robots

**DOI:** 10.3390/biomimetics10070435

**Published:** 2025-07-02

**Authors:** Yongjiang Xue, Wei Wang, Mingyu Duan, Nanqing Jiang, Shaoshi Zhang, Xuan Xiao

**Affiliations:** 1 School of Computer Science and Technology, Tiangong University, Tianjin 300387, China; xueyongjiang@tiangong.edu.cn (Y.X.); 2110310310@tiangong.edu.cn (N.J.); 2 Department of Electronic Information and Engineering, Tiangong University, Tianjin 300387, China; wangweibit@tgu.edu.cn (W.W.); mingy.duan@gmail.com (M.D.); 3 School of Control Science and Engineering, Tiangong University, Tianjin 300387, China; zhangshaoshi2003@163.com; 4 Tianjin Key Laboratory of Autonomous Intelligence Technology and Systems, Tianjin 300387, China

**Keywords:** wheel-legged robots, rolling gait, swing gait, specific resistance

## Abstract

Legged robots face inherent challenges in energy efficiency and stability at high speeds due to the repetitive acceleration–deceleration cycles of swing-based locomotion. To address these limitations, this paper presents a motion strategy that uses rolling gait instead of swing gait to improve the energy efficiency and stability. First, a wheel-legged quadruped robot, R-Taichi, is developed, which is capable of switching to legged, wheeled, and RHex mobile modes. Second, the mechanical structure of the transformable two-degree-of-freedom leg is introduced, and the kinematics is analyzed. Finally, experiments are conducted to generate wheeled, legged, and RHex motion in both swing and rolling gaits, and the energy efficiency is further compared. The experimental results show that the rolling motion can ensure stable ground contact and mitigate cyclic collisions, reducing specific resistance by up to 30% compared with conventional swing gaits, achieving a top speed of 0.7 m/s with enhanced stability (root mean square error (RMSE) reduction of 22% over RHex mode). Furthermore, R-Taichi exhibits robust multi-terrain adaptability, successfully traversing gravel, grass, and obstacles up to 150 mm in height.

## 1. Introduction

Legged robots, including bipeds, quadrupeds (e.g., ANYMal [1,2,3], Mini-Cheetah [4]), and hexapods, have been extensively studied and applied in various fields such as search and rescue, environmental monitoring, and industrial tasks. Inspired by biomimicry, most legged robots utilize a swing mechanism, achieving stable and efficient walking gaits. However, the swing mechanism requires repeated acceleration and deceleration of the legs, leading to significant energy loss at high speeds. Moreover, the swing motion imposes stringent requirements on leg inertia, joint torque, and response frequency, which limits the speed of locomotion.

The rimless wheel walker [5]), developed by McGeer, employs passive dynamic walking to achieve a rolling gait. Subsequently, an increasing number of rolling gait robots have been proposed, such as the RHex [6] series robots, including RHex-T3 [7] and Q-Whex [8], which implement rolling gaits through different approaches. Later, the robots include the Whegs series [9,10,11,12,13] by R.E. Ritzmann et al. and ASGUARD [14] by Frank Kirchner, which simplified control systems and generated more stable gaits. Notably, these robots demonstrate energy consumption levels intermediate between wheeled and legged systems. These robots utilize the rotational motion of their legs for locomotion. Each leg is independently actuated and sequentially contacts the ground to propel the robot forward, effectively emulating wheeled propulsion. This gait enables the robots to move efficiently across complex terrains such as grass, sand, and rocky surfaces, while demonstrating exceptional obstacle negotiation and stair-climbing capabilities. Recent advances in hybrid locomotion design have demonstrated promising approaches for terrain adaptation. While transformable mechanisms like Chen’s leg–wheel module [15] enable rapid mode switching, their energy efficiency comparison remains unexplored. Similarly, hexapod designs such as Ju’s Chebyshev-linkage robot [16] optimize payload capacity but focus solely on legged motion. In addition, several other studies have also made significant contributions to the field of wheel-legged robots. For instance, the wheel-legged robot [17] based on an adaptive sliding mode controller (ASMC) and linear quadratic regulator (LQR) significantly enhances the robot’s mobility and disturbance rejection on complex terrains. Another wheel-legged robot [18] with hierarchical optimization improves efficiency and stability in complex environments. Additionally, the combination of linear quadratic regulator (LQR) and active disturbance rejection controller (ADRC) in robots [19], through dual-loop control strategies, greatly enhances the robustness of wheel-legged robots. Meanwhile, a hydraulically driven bipedal wheel-legged robot [20] has been proposed, achieving stable jumping motions and precise control of jump height and distance. These studies collectively advance the performance optimization of wheel-legged robots in terms of control strategies and terrain adaptability.

Nevertheless, the robot’s legs undergo significant periodic rigid collisions with the ground surface, leading to unavoidable energy dissipation. Meanwhile, such rigid collisions will also reduce the robot’s stability and cause relatively serious damage to the robot’s thigh and leg joints, thereby shortening the robot’s service life. To address these challenges, Lin et al. enhanced this mechanism by introducing knee joints and proposed a series of wheel-legged hybrid structures [21,22] that generate rolling gaits. Rolling gaits combine the efficiency and speed of wheeled systems with the stability of legged gaits. Compared with swing gaits, rolling gaits demonstrate reduced thigh speed fluctuations, which results in lower energy consumption and higher speeds. However, these robots still require further research to optimize their performance.

Therefore, in this article, we developed a wheel-legged robot, R-Taichi, to analyze the gait properties of the swing and rolling mechanisms. It is equipped with the fundamental capability to perform active gait control through articulated knee joints, enabling programmable alternation between the stance and swing phases. Unlike passive wheel deformation, the leg mechanism actively regulates ground interaction through precisely controlled joint movements, which is demonstrated in the subsequent sections on gait generation analysis and stability metrics. The robot’s legs incorporate a two-degree-of-freedom linkage mechanism, enabling the robot to achieve the functions of rolling and swinging. Based on this design, we implement traditional wheeled locomotion, legged locomotion, and RHex-style locomotion. Additionally, by combining the advantages of the swing gait and the rolling gait, we propose a novel roll-leg motion strategy to enhance the energy efficiency and stability of the legged locomotion. By switching the motion control strategy, R-Taichi achieves efficient locomotion across various terrains and environments, reducing energy consumption while improving both stability and speed.

In summary, the contributions of this work are as follows:

1. A novel hybrid locomotion strategy was developed, combining rolling and swinging movements to overcome limitations inherent in both legged and RHex-type locomotion. This hybrid strategy was implemented using trot and walk gaits. The feasibility of this approach was validated through stability and power consumption experiments, which also revealed the relative advantages and disadvantages of the various motion modes. These findings offer new insights for the design of future motion strategies for wheel-legged robots.

2. A transformable wheel-legged quadruped robot was developed. Its wheels can be deformed to enable both traditional rolling, swinging, and jumping motions, combining these gaits to allow the robot to switch among wheel mode, leg mode, and RHex mode, adapting to different motion environments. The terrain adaptability and efficiency of R-Taichi are demonstrated through a series of motion experiments. This design not only advances the application of rolling gaits but also provides new insights for enhancing robot performance in complex environments.

## 2. Mechanical Design

### 2.1. Overall Robot Architecture

The R-Taichi robot embodies a hybrid locomotion paradigm, combining the efficiency of wheeled movement with the adaptability of legged locomotion. This synthesis addresses the speed–stability trade-off prevalent in conventional mobile robots through its transformable wheel–leg mechanism.

**Figure 1:**
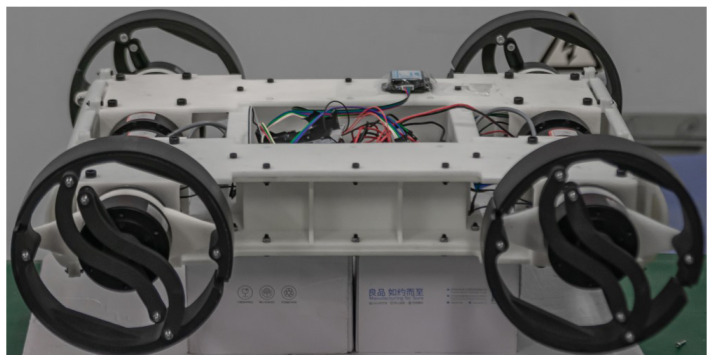
Physical structure of R-Taichi.

**Figure 2:**
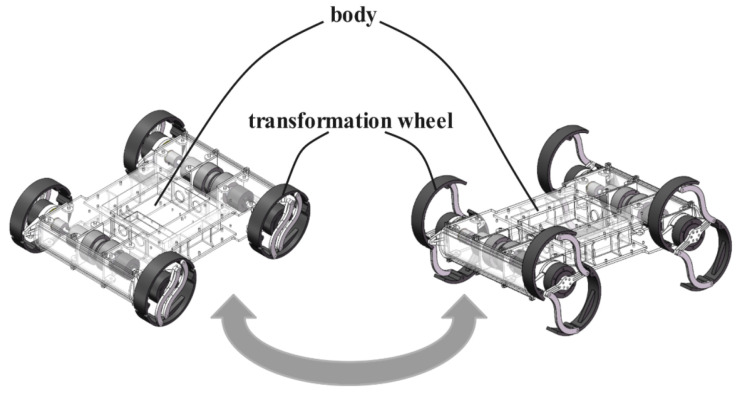
Overall description of the robot and leg transformation.

As illustrated in Figure 1, the robot features a square chassis (580 × 490 × 180 mm) with four 2-DOF transformable limbs arranged in bilateral symmetry. The quasi-hollow structure reduces weight while accommodating control electronics, achieving a 10.6 kg total mass through optimized material distribution. The six-bar linkage leg mechanism achieves seamless transition between circular (wheeled) and semicircular (legged) configurations through coordinated motor control, enabling the robot’s dual-mode locomotion capability as shown in Figure 2. Dual-motor architecture decouples locomotion and transformation functions (see Section 2.2). Conductive slip rings prevent cable entanglement during continuous rotation.

### 2.2. Transformable Leg Mechanism

#### 2.2.1. Mechanical Implementation

**Figure 3:**
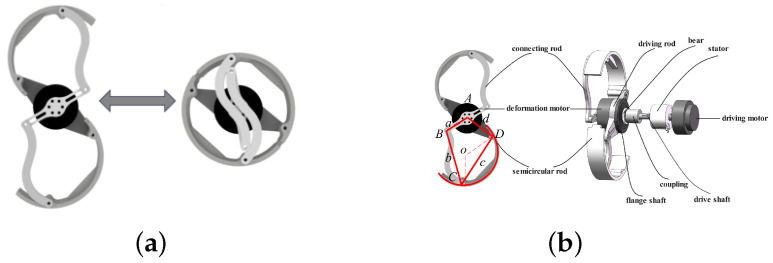
Structural layout of the leg mechanism. (**a**) Overview of the wheel-leg transformation mechanism. (**b**) Detailed components of the transformation mechanism.

The transformable leg consists of motors and a linkage mechanism, as shown in Figure 3. The inner motion motor is connected to a long flange shaft, which is securely fixed and extends through a conductive slip ring to one end of the coupler. A key embedded in the flange shaft establishes a stable, fixed connection at this interface. The opposite end of the coupler is linked to a larger flange shaft, which is secured in a similar manner and connects to the drive rod.

The robotic leg also incorporates an external transformation motor, which drives the leg’s transformation through a linkage mechanism. This motor is mounted on a crank, which is hinged to both an S-shaped rod and a semicircular rod, enabling controlled leg Transformation. The rotation of the Transformation motor activates this mechanism, allowing the leg to adapt its structure accordingly.

The transformable wheel consists of a transformation motor and two symmetric four-bar linkages, which functionally operate as a six-bar mechanism. The rotor of the transformation motor is rigidly coupled to a crank, which, through an ‘S’-shaped connecting rod, is linked to the semicircular leg. The rear of the Transformation motor is affixed to a drive rod and a flange disk using screw holes. These components are then connected to the robot’s internal motion motor via a drive shaft. The drive shaft is fitted with a coupler and a conductive slip ring, the function of which has been described previously.

When the crank angle is set to zero degrees (configurable), the robot’s leg reaches its maximum opening angle. To ensure proper alignment of the crank and connecting rod as the wheel completes its rotation, the connecting rod is designed with an ‘S’ shape. This configuration allows the two components to remain in phase during motion, ensuring smooth operation. Additionally, the semicircular rod is designed with a shorter arc on the side that interfaces with the input link of the motion motor. This arc is equipped with a groove to prevent interference with the crank, enabling uninterrupted movement of both the wheel and the leg.

This design prevents mechanical collisions during operation, ensuring that both the wheel Transformation and leg movement can occur without mutual interference. The seamless interaction between these components allows for efficient leg transformation and smooth transitions between the robot’s different modes of locomotion.

**Figure 4:**
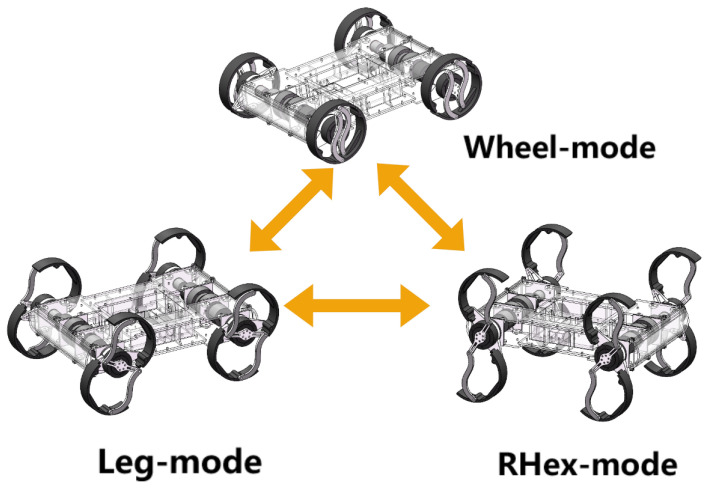
The strategy of switching mode.

As illustrated in Figure 3 and Figure 4, the transition from wheel mode to leg mode begins with the robot’s drive motor rotating to actuate the driving rod. This rod, through a connecting linkage, transfers motion to the semicircular rod, prompting it to unfold from a closed circular configuration into a leg-like structure. When the semicircular rod reaches the predetermined angle of 90°, the drive motor locks into this position to secure the mechanism, thereby ensuring stability in leg mode. Subsequently, the robot switches to discrete gait control, which allows for independent leg actuation. This independent actuation is crucial for crawling or negotiating obstacles, enabling the robot to adapt to complex terrains. The transition from wheeled mode to legged mode, as well as from RHex mode to legged mode, requires appropriate timing to ensure that the supporting leg can maintain the balance of the robot.

#### 2.2.2. Conductive Slip Ring Integration for Wire Management

A key feature of this design is the use of wired signal transmission. The control of the four motors on each side of the robot is integrated into a single communication bus, which interconnects both internal and external motors, carrying both signal and power lines. Without proper management, this configuration could lead to wire entanglement during cyclic motion.

To address this issue, a conductive slip ring is incorporated. The wires from the motion motor, routed from the internal PCB, enter the slip ring’s stationary stator and exit through its rotating rotor to connect to the Transformation motor. As the motors operate, only the rotor-connected wiring rotates with the shaft, while the stator remains stationary. By securing the rotor wires along the rotating shaft, wire entanglement is effectively prevented.

This approach resolves the issue of wire entanglement and contributes to a more manageable control architecture. Additionally, decoupling the motion and transformation functions of the motors ensures that their operations remain independent, simplifying control strategies under various movement conditions and enhancing the robot’s efficiency and reliability.

### 2.3. Experimental Platform

#### 2.3.1. Physical Configuration

To reduce the robot’s weight while maintaining cost-effectiveness, the chassis is constructed using a resin material, which is fabricated through 3D printing. This approach not only minimizes weight but also allows for complex geometries to be realized at a lower cost. The robot’s legs, which are essential for load-bearing and subject to wear during locomotion, are made from high-strength nylon. This material choice ensures the necessary durability and structural integrity to withstand the stresses encountered during dynamic movement.

The MyActuator RMD-X6 servo is employed for actuation, delivering the required high torque and rapid rotational speeds to meet the robot’s dynamic performance demands. This model is specifically selected for its proven reliability in executing demanding maneuvers, including leg transformation and seamless motion transitions.

Table 1 presents the detailed parameters related to the robot’s construction and its deformable leg mechanism.

#### 2.3.2. Control System

**Figure 5:**
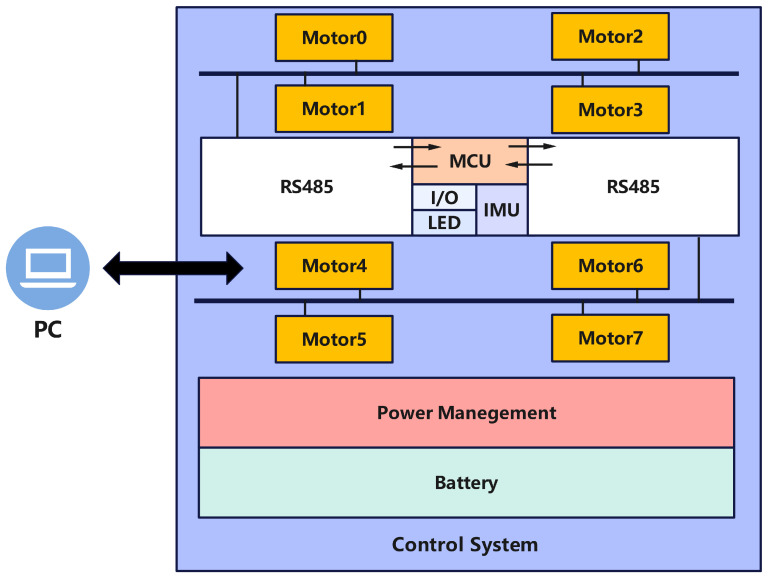
Control system block diagram.

The control platform consists of the power supply, control system, communication bus, and sensors, as shown in the system block diagram in Figure 5. The power supply is divided into two separate units. The first unit powers the motors, utilizing a 6S RC lithium battery that distributes power to the eight servo motors via a custom-designed power distribution board. The second unit powers the control board, using an 18,650 lithium battery to supply power to the main control board and associated external sensors.

The control system consists of the main control subsystem and several peripheral modules. The core of the system is the STM32F401RCT6 microcontroller (STMicroelectronics, Geneva, Switzerland), which is mounted on the control board. Peripheral modules include two TTL-to-RS485 bidirectional communication modules (ZY-485, Zhiyuan Electronics, Shenzhen, China), a wireless communication module, and an Inertial Measurement Unit (IMU). The system employs dual RS485 communication buses (RS485B, Waveshare, Shenzhen, China), each connecting four motors per side. These buses interface with the TTL-to-RS485 modules on the control board, which then communicate with two serial ports on the main control board. The sensor suite consists of the IMU and integrated sensors within the motor drivers. The IMU measures the robot’s three-axis tilt angles and acceleration, while the embedded sensors within the servo motors monitor torque, current, and rotational speed.

## 3. Kinematic Analysis

**Figure 6:**
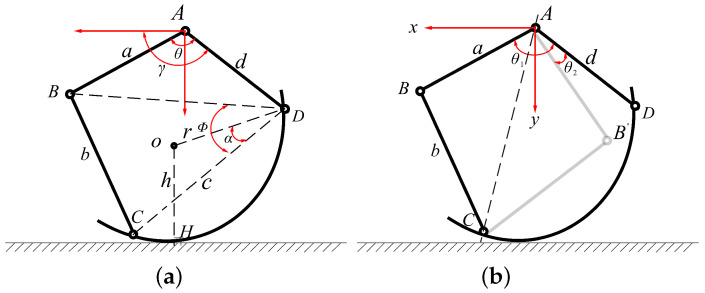
Kinematics analysis diagrams of the linkage mechanism. (**a**,**b**) illustrate the definition of kinematic parameters.

Although the structure of the transformable leg decouples rotation and Transformation movements, precise legged locomotion can still be achieved through accurate forward and inverse kinematics control. Figure 6 shows a kinematic analysis diagram of the linkage mechanism and its embodiment on a schematic of the robot leg.

### Forward Kinematics and Inverse Kinematics Analysis

For this analysis, we consider half of the linkage mechanism and assume ideal contact with the ground at a location. The forward kinematics problem involves determining the coordinates of this point $\left(H_{x},H_{y}\right)$, given the relative rotation angle θ of the crank AB and the rotation angle γ of the driving rod AD. To solve this, we apply the vector method. By establishing a Cartesian coordinate system with the fixed point A on the robot body as the origin, we derive

(1) AB+BC=AD+DC.

Decomposing the vectors along the orthogonal coordinate system, since the semicircular rod should always point in the positive x-axis direction, we can derive the direction angle of DC using trigonometric substitution:

(2) $$\phi =\arctan\frac{\left(E-G)*(F-L\right)}{\left(E-G-F+L)*(E-G+F-L\right)}.$$

where

(3) $$\begin{array}{c} \left\{\begin{array}{c} E=d*\cos\gamma -a*\cos\left(\gamma -\theta \right),\;\\ F=d*\sin\gamma -a*\sin\left(\gamma -\theta \right),\;\\ G=\frac{E^{2}+F^{2}+c^{2}+b^{2}}{2c},\;\\ L=\sqrt{E^{2}+F^{2}-G^{2}}. \end{array}\right. \end{array}$$

Next, considering the nature of the tangent point, we have (OH) parallel to the y-axis, and we formulate and solve the second vector equation:

(4) AH+AD=DO+OH.

Since the position of the center relative to the semicircular rod is fixed, we have

(5) $$\cos\alpha =\frac{c}{2r}.$$

Given that the direction angle of DO is ϕ−α, we obtain

(6) $$\left[\begin{array}{c} H_{x}\\ H_{y} \end{array}\right]=d\left[\begin{array}{c} \cos\gamma \\ \sin\gamma \end{array}\right]+r\left[\begin{array}{c} \cos(\phi -\alpha )\\ \sin(\phi -\alpha ) \end{array}\right]+h\left[\begin{array}{c} 0\\ 1 \end{array}\right]$$

where h is the distance from the outer arc to the center, considering the part thickness.

By solving the forward kinematics and using the position parameters returned by the motor encoders during leg movement, we can determine the reachable positions of the linkage end for given angles θ and γ, i.e., the workspace of the leg. This allows us to determine the angle conditions required for specific positions, which are the conditions for the existence of inverse kinematics solutions. We use MATLAB (version R2023a) to iterate through the motor rotation angles θ and γ. We specify that when CH·DH≥ and ∥CH∥ ≥ ∥EH∥, the tangent point H is outside this workspace. This is because, under these conditions, the tangent point will fall within the arc CE, which is unfavorable for any motion mode.

**Figure 7:**
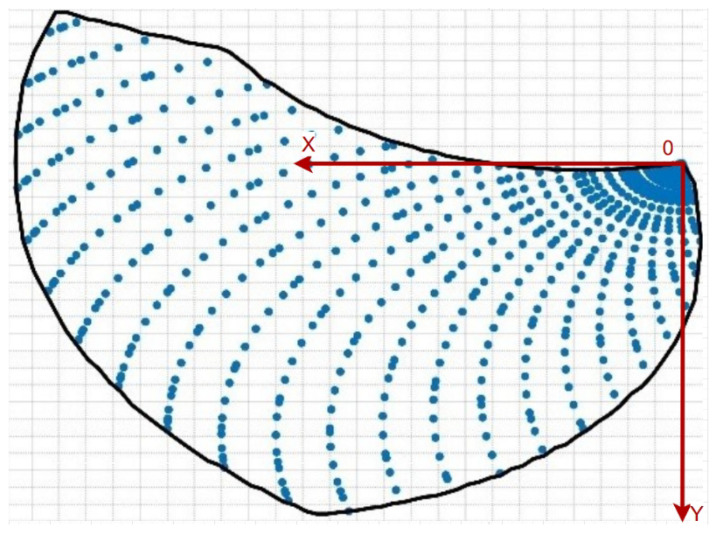
R-Taichi leg’s movement space.

As shown in Figure 7, the horizontal (X) axis is defined along the direction of the robot’s body, and the vertical (Y) axis is defined perpendicular to the direction of the robot’s body, both with reference to the robot itself. The area within the black line represents the theoretical workspace of the leg. However, due to factors such as robot weight, terrain, and motor torque limits, the actual workspace of the leg will be a subset of the theoretical workspace.

The details of the inverse kinematics solution are not elaborated here and are summarized as follows: the joint angle trajectories are generated by an optimized inverse kinematics solver that incorporates both mechanical constraints and terrain geometry. This solution enables smooth gait transitions while effectively avoiding singular configurations.

## 4. Gait Mode Generation

Through the preceding analysis of forward and inverse kinematics, both wheel mode and legged mode motions were successfully implemented on the robot. Building on its multimodal locomotion capabilities, this section presents a detailed analysis of various gaits, including wheel mode, RHex mode, and traditional swing-legged modes such as trot and walk. We also compare these gaits with our proposed rolling-legged mode to evaluate their performance under similar conditions. Figure 2 shows the gait diagram and the trajectory of the locomotion point P on the legs.

### 4.1. Wheel Mode

In wheel mode, the robot’s legs fold into fully compact configurations, forming complete circular wheels. Each wheel is independently driven by its dedicated motion motor. Using closed-loop speed control, all four wheels can rotate synchronously at the same speed, allowing the robot to achieve linear motion in both forward and reverse directions. The robot’s velocity and travel distance are directly determined by the rotational speed of the motors.

### 4.2. RHex Mode

In RHex mode, R-Taichi achieves locomotion through leg rotation without the need for knee joint adjustments. During this mode, the robot’s legs are fully extended, and the Transformation motors remain inactive. Locomotion is driven by the motion motors, which alternate between rolling and stance phases. The stance phase, characterized by a slower swing speed, is followed by the rolling phase, which operates at a higher speed. The legs on diagonally opposite corners are synchronized, while those on the same side simultaneously undergo either the rolling or stance phase. The velocities of these phases, denoted as $V_{fast}$ and $V_{slow}$, are determined by the following equations:

(7) $$V_{fast}=\frac{r\cdot \phi_{f}}{t_{fast}}$$

(8) $$V_{slow}=\frac{r\cdot (\pi -\phi_{f})}{T_{R}-t_{fast}}$$

where $T_{R}$ represents the gait cycle period in RHex mode, *r* is the radius of the leg, $\phi_{f}$ is the fast swing phase angle, and $t_{fast}$ is the duration of the fast swing phase.

It is important to note that, due to the symmetric design of R-Taichi’s legs, only an angular displacement of π is required to complete a full cycle, unlike the traditional RHex robot. As a result, the stance angle in R-Taichi is doubled compared with that of the traditional RHex, theoretically enabling locomotion speeds twice as fast. Additionally, this mode reduces speed fluctuations during phase transitions, minimizing energy consumption and enhancing the stability of the robot’s movement.

### 4.3. Swing-Leg Trot Gait

The trot gait is a classic high-speed gait. In this gait, the diagonal legs move in phase, while the legs on the same side alternate between the stance and swing phases (swing phase for swing-leg mode and rotation phase for rolling-leg mode). Typically, in a trot gait, the duty cycle of the legs is 50%, as shown in Figure 2, which is also the gait used in this experiment.

In the swing-leg trot gait, the robot’s deformable wheels transition between stance and swing phases according to specific trajectory equations. The design of the trajectory equation for the swing phase is crucial, as it directly impacts the robot’s ability to cross obstacles, its stability, and its energy efficiency. The trajectory equation for the foot end during the swing phase is as follows:

(9) $$x\left(t\right)=E\left[\frac{t}{t_{\mathrm{sw}}}-\frac{1}{2\pi }\sin\left(2\pi \frac{t}{t_{\mathrm{sw}}}\right)\right]-\frac{E}{2}.$$

(10) $$\begin{array}{c} y\left(t\right)=\left\{\begin{array}{cc} \frac{2Ht}{t_{\mathrm{sw}}}-\frac{2H}{4\pi }\sin\left(4\pi \frac{t}{t_{\mathrm{sw}}}\right) & t<\frac{t_{\mathrm{sw}}}{2},\;\\ 2Ht-\frac{2Ht}{t_{\mathrm{sw}}}+\frac{2H}{4\pi }\sin\left(4\pi \frac{t}{t_{\mathrm{sw}}}\right) & \frac{t_{\mathrm{sw}}}{2}<t<t_{\mathrm{sw}}. \end{array}\right. \end{array}$$

These equations, when combined with the foot-end trajectory graph, reveal that the robot achieves a smooth and continuous foot motion, ensuring a controlled and soft landing. This design not only minimizes impact forces but also enhances the overall stability of the robot during locomotion, enabling it to navigate challenging terrains with greater precision and balance.

### 4.4. Swing-Leg Walk Gait

The walk gait is a standard slow gait, distinguished by its emphasis on stability. In leg mode, when operating in the walk gait, the diagonal or same-side legs do not necessarily move in phase. This is due to the leg duty cycle being set to 25%, as shown in Figure 2. As a result, in each sub-cycle, three legs remain in the stance phase, providing increased stability at low speeds compared with the trot gait.

In swing-leg mode, we implemented both the trot and walk gaits. The trot gait, with its shorter stance phase, achieves higher speeds and is more suitable for dynamic movement. In contrast, the walk gait prioritizes stability, making it ideal for steady, controlled locomotion.

### 4.5. Rolling-Leg Trot Gait

**Figure 8:**
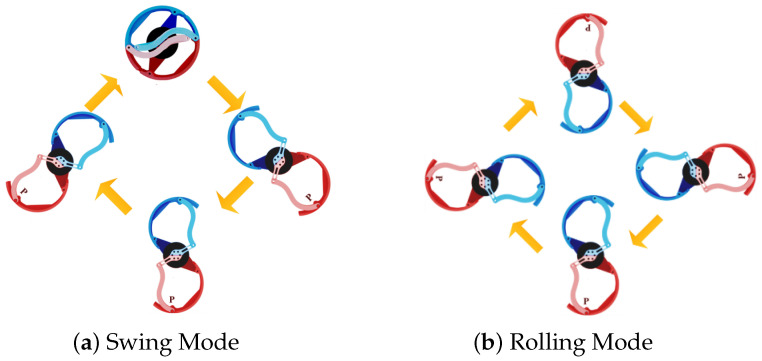
The distinct locomotion modes of our robotic system. (**a**) the swing mode, showing the motion process of the leg mechanism, and (**b**) the rolling mode, depicting the motion process of the leg mechanism.

Previous analysis indicates that the traditional swing-leg mode places significant load on both the motors and the robot during high-speed movement. In the rolling-leg strategy, the swing phase of the airborne state is converted into rotational motion, as shown in Figure 8. To maintain symmetry in the movement cycle, the motion motor must rotate 180° after each swing and rotational phase. Therefore, each complete cycle consists of two rotational phases and two swing phases.

Due to the design of our leg structure, the knee adjustment height during the stance phase is minimal, requiring only a small rotation angle for the Transformation motor, which has a negligible impact on the angular velocity of the motion motor. During the rotational phase, the Transformation motor remains stationary relative to the motion motor. As a result, the rotation of the motion motor plays a dominant role in both energy consumption and speed. The relationship for the motion motor is given by

(11) $$\frac{\omega_{r}}{\omega_{s}}=\frac{\theta_{r}*t_{s}}{\theta_{s}*t_{r}}$$

(12) $$\theta_{r}+\theta_{s}=\pi$$

where $\theta_{s}$ and $\theta_{r}$ represent the angles of the Transformation motor during the stance and rotational phases, respectively. The variables $t_{s}$ and $t_{r}$ denote the durations of the stance and rotational phases, while $\omega_{s}$ and $\omega_{r}$ correspond to the angular velocities in the stance and rotational phases, respectively. In the rolling-leg trot, the duty cycle of the robot’s legs is set to 50%, resulting in equal durations for the stance and swing phases. The ratio of angular velocities is directly proportional to the ratio of rotation angles. Minimizing speed variation between these phases is hypothesized to reduce unnecessary energy consumption in the motors and enhance the robot’s stability during movement. When the rotation angles are equal, the relationship $\omega_{s}/\omega_{r}=1$ is achieved. This suggests that in trot mode, controlling the leg rotation angle enables regulation of the angular velocity ratio between the stance and swing phases. Specifically, when both θ angles are 90°, the angular velocities in both phases are approximately equal and uniform. This approach promotes nearly continuous angular velocity during trot movement, which is advantageous for reducing energy consumption during state transitions.

### 4.6. Rolling-Leg Walk Gait

In the walk gait, the duty cycle is set to 25%, resulting in a stance phase that lasts three times longer than the swing phase. Since the rotation angle during the stance phase cannot be excessively large, both angles are assumed to be 90°. Based on the previous analysis, the relationship $\omega_{s}/\omega_{r}=3$ is derived, indicating a significant variation in speed between the stance and swing phases.

Figure 8 illustrates the distinct locomotion modes of our robotic system: (a) The swing-leg mode demonstrates programmed pendulum motion, where the leg swings forward to its maximum position before abruptly stopping and reversing direction to complete the rear swing phase; this characteristic acceleration/deceleration pattern creates discrete ground contact transitions. (b) In contrast, the rolling-leg mode achieves unidirectional rotational movement, eliminating the stop–reverse motions of the swing mode and maintaining continuous ground contact, thereby enhancing motion smoothness while improving both energy efficiency and stability.

## 5. Experimental Tests

As previously described, the leg structure and body layout of R-Taichi enable various movement modes. This section presents experiments conducted on wheel mode, RHex mode, and leg mode. In leg mode, experiments were performed using both the traditional swing-leg mode and the newly proposed rolling-leg mode. The experimental gaits in leg mode included the trot and walk gaits. Data were collected to evaluate energy consumption and torque, and the results were analyzed to assess performance.

### 5.1. Energy Efficiency of Different Gaits

**Figure 9:**
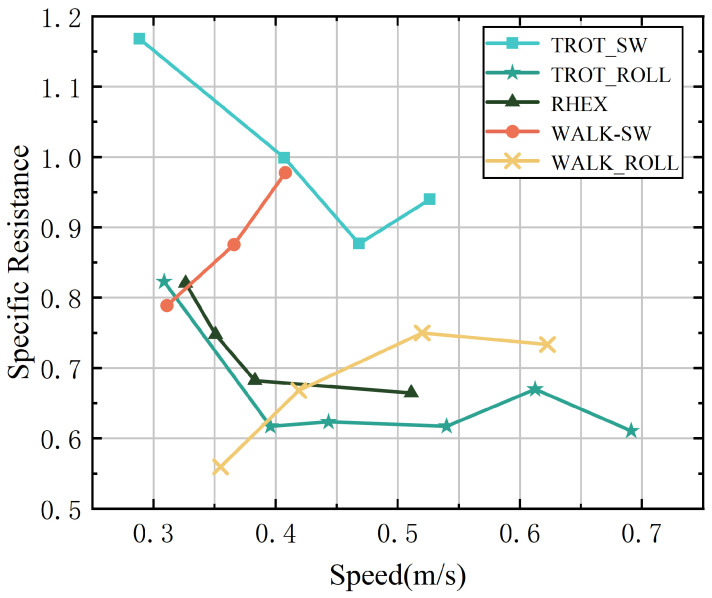
Specific resistances of the robot while running in five different gaits/modes.

We analyzed the gait characteristics of different modes and motion strategies from the perspectives of energy consumption and torque. Specific resistance (SR) was used as the metric for energy consumption, calculated by the following formula:

(13) $$SR=\frac{P}{mgv}$$

where *P* represents the average power consumption for each mode, taken as the robot’s total power consumption, mg is the robot’s weight at rest, and *v* is the robot’s average speed in each experiment. Each mode was tested at various speeds on a marble floor, with average power consumption measured using a power meter. Figure 9 presents the SR curves for different modes as a function of speed. The following analysis evaluates the performance of different strategies and modes based on these curves.

**Rolling-Leg Trot Gait**—The swing-leg walk gait is well suited for low-speed movement, offering high stability and low energy consumption to maintain balance at these speeds. However, as speed increases, energy consumption rises significantly. This increase is mainly due to the additional force required to maintain stability at higher speeds. As the speed continues to rise, the high load imposed on the body and motors from rapid leg swing limits the effectiveness of the walk gait. This explains the limited data points for this gait in the SR graph and the observed increase in energy consumption with speed.

**Rolling-Leg Walk Gait**—Compared with the swing-leg walk gait, the rolling-leg walk gait offers a significant improvement in energy efficiency. This strategy maintains low energy consumption at low speeds, with a noticeable increase as speed rises. By converting the swing phase into rolling motion during the duty cycle, the continuity of speed during phase transitions is enhanced. This improvement results in a higher maximum speed for the robot in this gait.

**RHex Mode**—RHex mode exhibits higher energy consumption at low speeds due to the instability of the center of mass. However, as speed increases, energy consumption decreases somewhat, as RHex mode stabilizes the center of gravity at higher speeds. This stabilization allows more energy to be used efficiently for locomotion.

**Swing-Leg Trot Gait**—The swing-leg trot gait shows higher specific resistance at low speeds due to the inherent instability of trotting at these speeds. As speed increases, energy consumption decreases, indicating that the trot gait is more efficient for higher-speed movement. Similar to the swing-leg walk gait, the maximum speed in this gait is constrained by the high load experienced during rapid leg swing.

**Rolling-Leg Trot Gait**—The rolling-leg trot gait exhibited superior performance in the tests, effectively balancing high speed and low energy consumption. At lower speeds, it consumed less energy compared with RHex mode, owing to the efficiency of knee adjustments. The continuous speed during phase transitions, facilitated by equal stance and rolling angles, further reduced energy consumption at higher speeds. This led to the highest speed and lowest energy consumption observed in the experiments.

In summary, the rolling-leg strategy demonstrates higher energy efficiency and enables greater maximum speeds compared with the swing-leg strategy in both walk and trot gaits. The energy consumption curve of the rolling-leg strategy exhibits distinct characteristics compared with the RHex mode. Furthermore, the swing-leg trot strategy consistently shows lower specific resistance than RHex mode, suggesting that it may be a more efficient motion strategy in terms of energy consumption.

**Figure 10:**
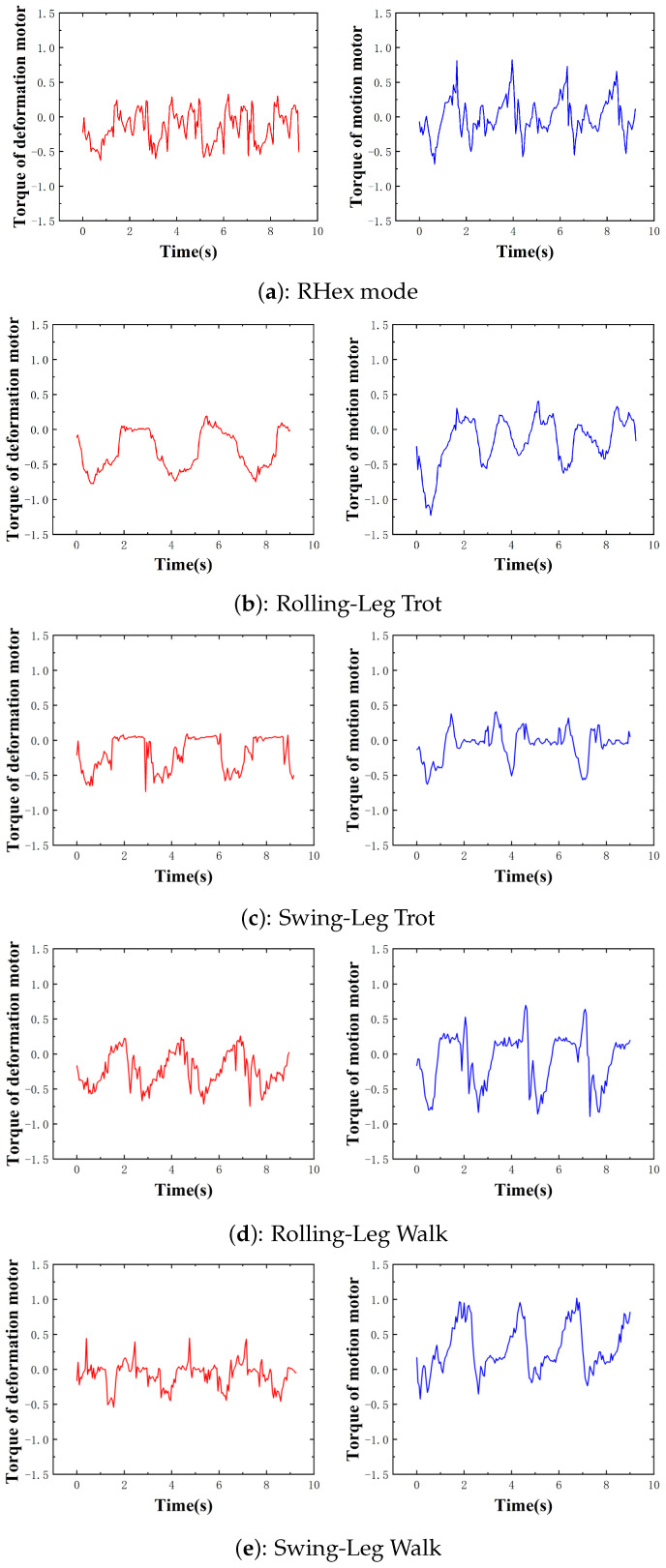
Torque curves of transformation motor and motion motor in different modes.

We plotted the torque curves of the motion and Transformation motors under different motion strategies at comparable speeds (approximately 0.4 m/s), as illustrated in Figure 10. This analysis focuses on comparing the traditional swing-leg mode with the rolling-leg strategy.

For the motion motor, the torque magnitude and variation during the stance phase are comparable across both strategies. However, during the duty phase, the rolling-leg strategy exhibits torque oscillations around zero, with abrupt changes occurring only at the transitions between stance and rotation phases. This behavior is attributed to the rolling motion in the duty phase, which minimizes frictional resistance and reduces the current required to generate driving torque.

In contrast, the swing-leg strategy necessitates rapid acceleration to execute the swing motion during the duty phase. As a result, the peak torque during this phase is significantly higher than that of the rolling-leg strategy, and the torque does not stabilize around zero. Instead, the motor must continuously overcome the momentum from the previous state, leading to increased energy losses and mechanical wear on the robot.

Similar trends are observed for the Transformation motor. During the stance phase, the torque output is comparable between the two strategies. However, in the duty phase, the rolling-leg strategy reduces the Transformation motor’s torque output to near zero, as only the motion motor is active. Conversely, the swing-leg strategy requires the Transformation motor to alternately close and open the leg, leading to higher energy consumption, especially at increased speeds.

In summary, the rolling-leg strategy demonstrates superior energy efficiency for both the motion and Transformation motors. At higher speeds, the swing-leg strategy produces more erratic torque patterns, as the motor must continuously accelerate and decelerate the leg. When the motor’s torque limit is exceeded, the swing-leg motion becomes unstable, resulting in distorted torque waveforms. This explains the lower maximum speed achievable in swing-leg mode compared with rolling-leg mode. The rolling-leg strategy benefits from consistent torque output throughout the motion cycle, enabling higher maximum speeds and reducing mechanical stress on the robot.

In RHex mode, the duty phase also involves rolling motion, resulting in torque characteristics similar to those of the rolling-leg strategy. However, during the stance phase, RHex mode lacks the buffering provided by knee extension and contraction in the rolling-leg strategy. Consequently, both the motion and Transformation motors in RHex mode generate higher torques during the stance phase compared with the rolling-leg strategy. This distinction in torque output highlights the primary difference between RHex mode and rolling-leg mode.

**Figure 11:**
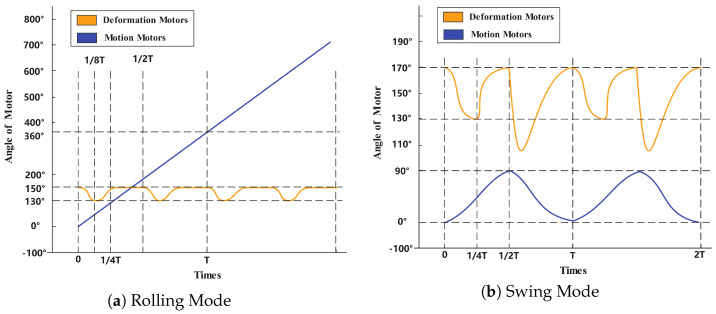
Rotational angle variation under different motion strategies. (**a**) shows the rotational angle variation in rolling mode, (**b**) shows the rotational angle variation in swing mode.

Figure 11 presents a comparative analysis of the angular displacement profiles for both the motion motor and deformation motor across different locomotion strategies, derived through inverse kinematic computations based on the robot’s prescribed trajectory. The key observation reveals fundamentally distinct actuation patterns: under the rolling strategy, the primary motion motor exhibits a quasi-linear angular progression, demonstrating near-constant velocity characteristics that ensure smooth kinetic energy transfer. In contrast, the swing strategy induces pronounced periodic fluctuations in the motion motor’s angular displacement, corresponding to the repetitive acceleration-deceleration cycles inherent to pendulum-like leg motion. These quantitative differences mechanistically explain the rolling strategy’s superior velocity continuity and energy efficiency. The deformation motor’s angular profiles further corroborate this distinction, showing minimal variation during rolling versus active modulation in swing mode to facilitate leg repositioning.

### 5.2. Stability

To better illustrate the differences between the rolling-leg mode and the RHex mode, we also examined their stability in terms of body balance. We used the root mean square error (RMSE) to assess stability, defined as

(14) $$RMSE_{total}=\sqrt{{\left(RMSE_{\alpha }\right)}^{2}+{\left(RMSE_{\beta }\right)}^{2}}.$$

where

(15) $$RMSE_{\alpha }=\sqrt{\frac{1}{n}\sum_{i=1}^{n}{\left(\alpha_{i}-\alpha_{ref}\right)}^{2}},$$

and

(16) $$RMSE_{\beta }=\sqrt{\frac{1}{n}\sum_{i=1}^{n}{\left(\beta_{i}-\beta_{ref}\right)}^{2}}.$$

Since our robot is capable of crossing obstacles and navigating various terrains, we integrated pitch and roll angles to provide a comprehensive assessment of stability. In this context, α and β represent the roll and yaw angles of the robot’s forward direction, as recorded by the IMU. The variable $angle_{i}$ denotes the *i*-th measured tilt angle, while $angle_{ref}$ is the ideal stable tilt angle, assumed to be zero for simplicity. A smaller $RMSE_{total}$ value indicates smaller angle fluctuations, signifying more stable movement.

**Figure 12:**
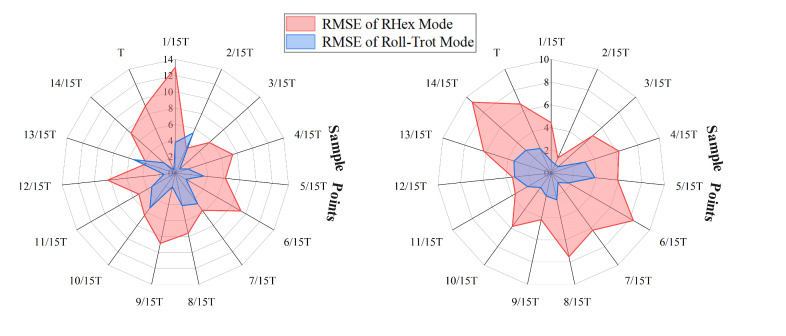
Comparison of RMSE between RHex mode and rolling-leg trot gait at speeds of 0.4 m/s (**right**) and 0.6 m/s (**left**). The encircled numbers 1 to 15 represent the number of sampling points.

To evaluate the stability of the robot under different locomotion modes, we collected the total root mean square error (RMSEtotal) values at various speeds, as shown in Table 2. The results indicate that the stability of the RHex mode slightly decreases with increasing speed, reflected by a moderate rise in RMSE values. Although this change is not drastic, it demonstrates the growing instability associated with pure rotational motion, where rapid changes in ground contact of the legs frequently shift the center of gravity. In contrast, the rolling-leg trot gait exhibits a notable trend of improved stability with increasing speed, with generally lower RMSEtotal values across all tested speeds. This suggests that incorporating knee joint adjustments and smoother transitions between stance and swing phases contributes to better posture control of the robot. To capture variations in body stability during the gait cycle, we sampled instantaneous RMSE values at 15 equally spaced points over a complete gait cycle, as illustrated in Figure 12. The figure presents a comparative analysis of instantaneous RMSE sampling data for the two locomotion modes. In the study, 15 sampling points per cycle were used, with the RHex mode represented by a pink curve and the rolling-leg trot gait by a blue curve. This sampling method comprehensively captures the stability characteristics of both modes throughout the motion cycle. The left and right panels correspond to performance metrics at different speeds: the left panel displays RMSE values at 0.6 m/s, while the right panel shows data collected at 0.4 m/s. The comparative analysis reveals that the rolling-leg trot gait (blue curve) consistently exhibits lower RMSE values than the RHex mode (pink curve) at both tested speeds. These findings provide empirical evidence for the superior dynamic stability of the rolling-leg trot gait under varying speed conditions.

This improvement can be attributed to the fact that, for quadruped robots, the pure rotation in RHex mode leads to frequent shifts in the center of gravity, thereby reducing stability. In contrast, the rolling mode, with additional knee joint adjustments, offers enhanced stability. The results highlight the effectiveness of the hybrid rolling–swing strategy in mitigating instability caused by abrupt momentum changes inherent in traditional RHex-style gaits.

Finally, we evaluated the robot’s multiterrain and obstacle-crossing abilities. The robot underwent testing on diverse terrains, including marble, grass, and gravel, and it successfully traversed each terrain type. Additionally, the robot demonstrated its capability to overcome obstacles with ease. By extending its legs to position the center of mass at 130 mm, it effortlessly traversed obstacles with a height of 150 mm.

### 5.3. Terrain Adaptability and Performance

#### 5.3.1. Insights on Robot Traversal Through Challenging Terrains

**Figure 13:**
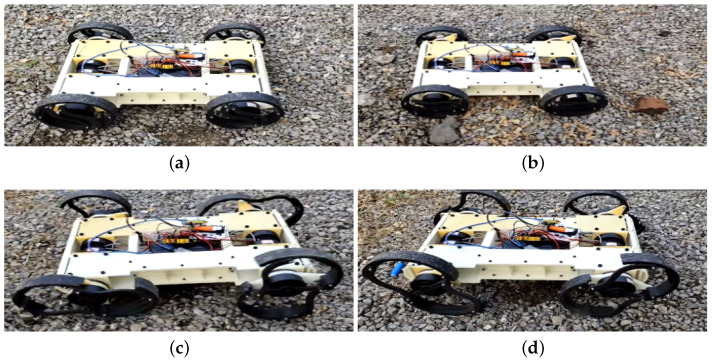
Locomotion Modes on Gravel Terrain. (**a**,**b**) show the wheeled mode of the robot on gravel terrain, while (**c**,**d**) depict the legged mode of the robot on gravel terrain.

For field robots, traversing challenging terrains, such as grasslands and gravel roads, is essential. Figure 13 demonstrates how R-Taichi navigates these surfaces using different locomotion modes. The first column of Figure 13 shows the robot employing a trot gait to traverse soft grass. The second column illustrates the robot navigating a hard gravel road. Notably, while traversing the gravel road, the robot utilizes both a simple wheeled mode and a more complex trot gait. This observation highlights that our robot is not only capable of adapting to various terrains but also exhibits robust performance with different locomotion modes on the same type of terrain.

#### 5.3.2. Obstacle Climbing by the Robot

**Figure 14:**
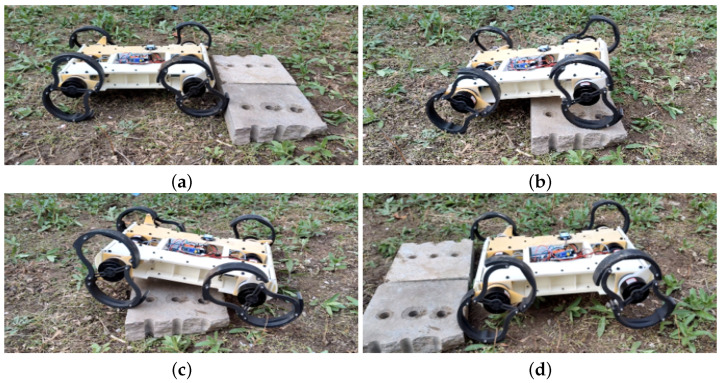
The sequence of images illustrates the robot’s process of overcoming an obstacle. Figures (**a**,**b**,**c**,**d**), respectively, depict the robot encountering the obstacle, approaching the obstacle, starting the crossing maneuver, and successfully crossing the obstacle.

We tested the obstacle-climbing capability of the robot. In Figure 14, the obstacle shown is a step with a height of 150 mm. The gait employed in this experiment is the traditional legged Trot gait, specifically the SW-Trot gait. As mentioned previously, thanks to the mechanical structure of R-Taichi, the length of its legs can be adjusted using Transformation motors. In the experiment shown in the figure, the leg height from the ground was set to 130 mm. It can be observed that the robot easily climbed over the obstacle without employing complex gaits or dynamic adjustment algorithms. Notably, the adjustable leg height of R-Taichi allows it to modify its climbing strategy based on the height of the obstacle.

#### 5.3.3. The Robot Traverses Cragged Gravel

**Figure 15:**
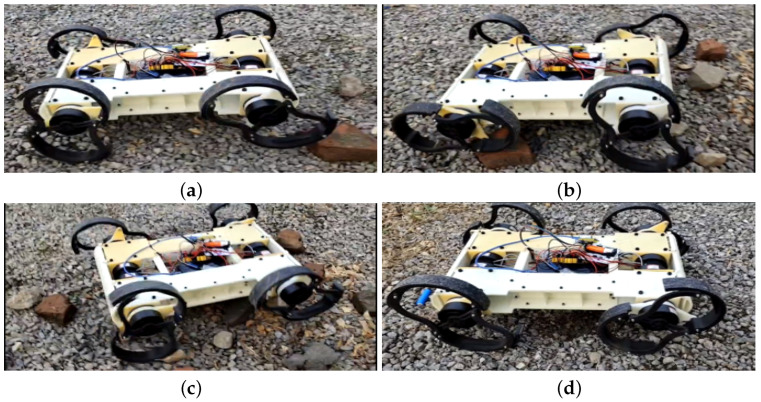
The images illustrating the robot traversing cragged gravel depict the following stages: (**a**) the robot encountering a small obstacle, (**b**) the robot’s rear wheels overcoming the obstacle, (**c**) the robot’s body positioned above the obstacle, and (**d**) the robot successfully passing the small obstacle.

One of the application scenarios for robots is performing tasks on rugged terrains, which serves as a standard for evaluating the challenges and capabilities of field robots. As shown in Figure 15, R-Taichi successfully accomplished this task. The terrain depicted in the figure is a cragged gravel road paved with obstacles such as broken bricks and stones. R-Taichi employed the RHex gait with its legs extended to a diameter of 220 mm, allowing it to easily traverse this type of terrain. During this process, the robot’s legs functioned like oversized wheels. This demonstrates the exceptional flexibility of R-Taichi’s leg control system.

### 5.4. Robot Jumping

#### Robot Jumping

**Figure 16:**
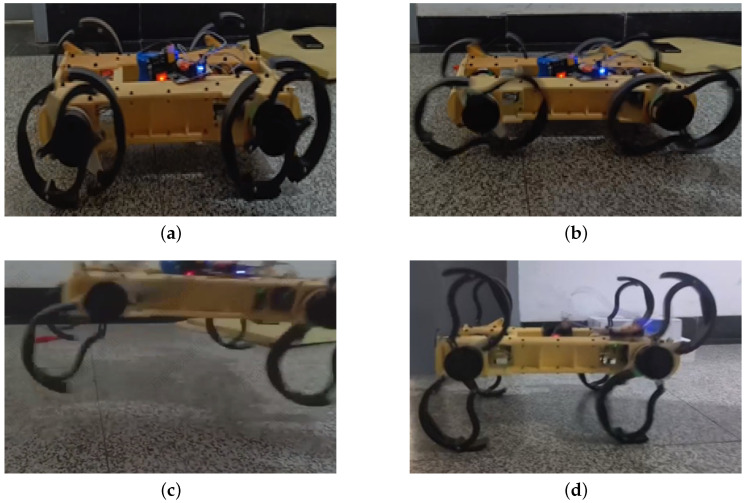
The robot’s jumping process is illustrated as follows: (**a**) initiation, (**b**) adjustment, (**c**) jumping, and (**d**) landing.

For a leg–wheel hybrid robot, performing basic gaits and obstacle traversal is a common task. However, achieving jumping capability in addition to these functionalities is not an easy feat. Nevertheless, R-Taichi has successfully accomplished this, as shown in Figure 16. The foundation of R-Taichi’s ability to jump lies in its deformable leg structure. Prior to jumping, the robot first prepares by moving in wheel mode. Just before the jump, the robot slightly extends its legs, as seen in Figure 16a. Following this, the robot’s legs suddenly extend from a small angle to a larger angle, much like a spring storing maximum elastic potential energy when compressed to its smallest size. We also aim for the robot to find the optimal angle and position for leg extension before the jump. Therefore, Figure 16b illustrates the adjustment process of the robot’s legs, a step that allows the robot to store as much potential energy as possible, which is provided by the motors. The next step involves the robot activating the leg motors. If only the deformable motor is activated, the robot will theoretically perform a vertical jump. However, if the drive motors are also activated, the robot can jump in other directions. As shown in Figure 16c, the robot performs a vertical jump. Finally, the robot lands, as shown in Figure 16d. This completes the jumping process. Currently, the highest jump achieved by the robot is 360 mm from the ground to the robot’s base.

## 6. Conclusions

This study presented the design, kinematic analysis, and experimental validation of R-Taichi, a novel hybrid wheel–leg robot inspired by the RHex platform. The robot demonstrates multimodal locomotion capabilities, transitioning between wheel mode, RHex mode, and multiple legged gait modes, including the proposed rolling-leg motion strategy. Through the analysis of forward and inverse kinematics, we established a framework for evaluating the performance of traditional and newly developed motion strategies under various gaits. The proposed rolling-leg strategy demonstrates significant performance advantages over conventional swing-mode locomotion, achieving a 30% improvement in energy efficiency by eliminating leg swing acceleration/deceleration cycles, and a 22% reduction in RMSE through continuous ground contact that enhances stability. These metrics are rigorously benchmarked against the robot’s own swing-mode operation. The experimental results highlight the energy efficiency and stability advantages of the rolling-leg strategy, particularly at higher speeds.

While the proposed rolling-leg strategy demonstrates significant advantages in energy efficiency and stability, several limitations should be noted. The current 10.6 kg platform weight restricts dynamic performance compared with lighter robotic systems, and the maximum speed of 0.7 m/s remains below that of wheeled counterparts. Our energy measurements focus primarily on mechanical systems without fully accounting for control electronics overhead. Additionally, while the experimental validation confirms the strategy’s effectiveness in controlled environments, further testing is needed to evaluate performance in more complex, unstructured terrains. These limitations highlight opportunities for improvement in powertrain optimization, comprehensive energy assessment, and robust control algorithms for highly dynamic scenarios.

Building upon the current findings, future research will focus on developing advanced control frameworks to enhance R-Taichi’s real-world applicability. Key directions include (1) intelligent gait-switching algorithms for adaptive locomotion across varying terrains, leveraging the demonstrated 360 mm vertical jumping capability for dynamic obstacle negotiation; (2) integrated perception-planning systems combining proprioceptive sensing for slip detection with motion optimization techniques; and (3) mechanical improvements through lightweight materials and 3D kinematic modeling to maintain structural integrity while improving mobility. These developments will specifically target search-and-rescue and exploration applications, where robust performance in unstructured environments is crucial.

## Figures and Tables

**Table 1:** Mechanical parameters of the robot.

Subject	Type	Parameter
R-Taichi	Body size	580 × 490 × 180 mm
	Material	nylon, resin-based
	Total weight	10.6 kg
	Instruction frequency	20 Hz
Leg	AB	6.2 cm
	BC	14 cm
	CD	13.8 cm
	AD	8.1 cm
Motor	Model	RMD-X6 1:8
	Nominal current	3.6 A
	Nominal torque	4.5 N·m
	Nominal output speed	310 rpm

**Table 2:** Stability Comparison between RHex Mode and Roll-Trot Mode.

Mode	ϵ in Different Speeds (m/s)		
	0.4	0.55	0.6
RHex	5.2	4.8	6.9
Roll-Trot	4.3	4.4	3.6

## Data Availability

The original contributions presented in this study are included in the article.
